# A Survey on Battery-Less RFID-Based Wireless Sensors

**DOI:** 10.3390/mi12070819

**Published:** 2021-07-13

**Authors:** Nabil Khalid, Rashid Mirzavand, Ashwin K. Iyer

**Affiliations:** 1 Intelligent Wireless Technology Lab., University of Alberta, Edmonton, AB T6G 2R3, Canada; nkhalid1@ualberta.ca (N.K.); mirzavan@ualberta.ca (R.M.); 2 Department of Electrical and Computer Engineering, University of Alberta, Edmonton, AB T6G 2W3, Canada

**Keywords:** battery-less, IoT, radio frequency identification (RFID), sensor, zero-power

## Abstract

We present a survey on battery-less Radio Frequency Identification (RFID-based wireless sensors that have emerged in the past several years. We discuss the evolution of RFID turning into wireless sensors. Moreover, we talk about different components of these battery-less RFID-based wireless sensors, five main topologies that transform a simple RFID chip into a battery-less wireless sensor, and state-of-the-art implementations of these topologies. In battery-less wireless sensors, the read range is of key importance. Hence, we discuss how each component of the sensor plays its role in determining the read range and how each topology exploits these components to optimize read range, complexity, and/or cost. Additionally, we discuss potential future directions that can help provide improvements in RFID-based wireless sensor technology.

## 1. Introduction

Wireless sensors are becoming increasingly popular in the home and industrial sectors and are used for a range of applications, from temperature or humidity monitoring to food-quality inspection of products being sold on the market. One of the main reasons for using wireless technology is that it affords non-contact, noninvasive sensing. This ability not only eliminates the need for long cables required for information transfer but also reduces the spread of germs and brings comfort to the users. To fully exploit the capabilities of wireless sensors and automatic processes, the future generation of wireless communication, 5G, and the evolving Industry 4.0 aims to incorporate them on a massive scale, due to which research on wireless sensors is on a dramatic rise [1,2,3].

For the past two decades, Radio Frequency Identification (RFID) has been widely used for object identification and tracking purposes [4]. It took many years for this technology to become affordable and reliable in a variety of applications. The technology was initially focused on replacing bar codes and Electronic Article Surveillance (EAS) to prevent theft. Later on, the application horizon of RFID became wider, and it was introduced in several applications such as tracking inventory at a warehouse for supply management purposes, automated toll collection without the need for stopping, and automatic unlocking of doors when entering parking structures or buildings premises [5,6].

As time passes, the applications of RFID are rapidly expanding, and RFID-based sensors are one of its most interesting applications. Initially, RFID technology was merely introduced for object identification. By the year 2004, the technology started incorporating sensing capabilities [7]. Although extending the chip’s capability from identification to sensing is straightforward, the design must ensure that the extension does not affect the performance of an RFID tag. Hence, such an addition should collect sufficient power to run the RFID chip and the sensing element without having any significant impact on the read range of the RFID tag [8,9].

**Figure 1:**
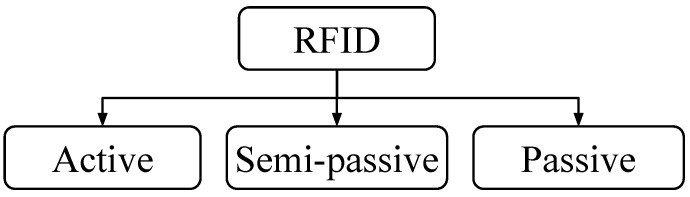
Taxonomy of RFID tags available on the market.

RFID tags are generally classified into three categories: active, semi-passive, and passive, as shown in Figure 1. An active RFID tag has its own power supply and a transmitter for communication, whereas a semi-passive RFID has its own power source but does not have any transmitter and, instead, uses a backscattering technique for establishing communication. On the other hand, a passive RFID chip, which is often the cheapest variety, has no internal power source and no transmitter and, thus, uses the power of the electromagnetic field transmitted by the reader to power-up its circuitry and to backscatter the received signal [10,11,12].

A sensing element may be incorporated in any of the aforementioned categories to design an RFID sensor. Using active or semi-passive technology requires a power source, which makes the wireless sensor bulky and expensive, whereas passive technology is much cheaper, but incorporating sensing elements in it is quite challenging due to the limited available power and flexibility. Hence, passive technology must be carefully engineered to address these challenges [3,11,13,14,15,16,17,18,19,20].

RFID is now a widely used technology for tracking and inventory management services and, as such, is governed by several design standards [21]. However, wireless sensors, especially RFID-based sensors, are still an emerging technology and, therefore, might be referenced using different names in the community. Particularly, passive wireless sensors are sometimes also termed battery-less, self-powered, or even zero-power [8,11,12,22,23,24,25,26,27,28].

**Figure 2:**
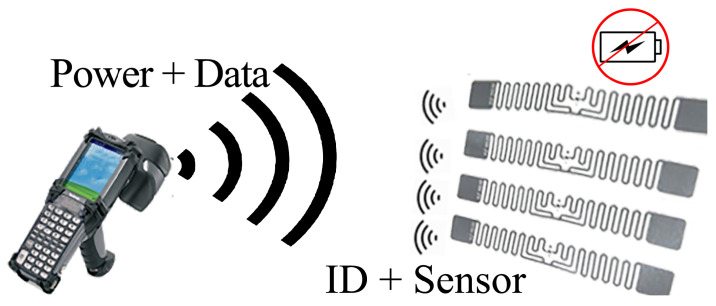
Battery-less wireless sensors draw energy from the reader and respond by backscattering the incident power signal.

Battery-less RFID-based wireless sensors have gained a lot of interest because they are lightweight, cheap, and long-lasting. In particular, the ones operating at ultra-high frequency (UHF) are of greater interest as they offer a good compromise between size and read range. An illustration showing the operation of battery-less wireless sensors is shown in Figure 2. There are several different designs proposed in the past that can be generally categorized into chip-less sensors, chip-based antenna resonance modifying sensors, multi-port chip-based sensors, digitally integrated sensors, and chip-based ambient energy-harvesting sensors. In the following sections, we discuss these categories in detail and observe the benefits and drawbacks of each [8,29,30,31,32,33,34,35,36,37,38,39,40,41,42,43,44,45,46,47,48,49].

The remainder of this paper is organized as follows. In Section 2, we discuss the individual components of RFID-based wireless sensors to develop a basic understanding of how they may be engineered to meet the requirements, e.g., complexity, cost, size, read range, and accuracy of a given application. In Section 3, the system topologies of different categories of battery-less RFID-based wireless sensors are discussed in the context of their complexity, cost, size, read range, and accuracy. Section 4 shows some examples of the implemented circuits that were selected based on their simplicity in design, completeness of the description in the article, price affordability, compactness in size, and adequate read range. The section also presents techniques to test each topology. Finally, potential future directions are presented in Section 5, and then, the paper is concluded in Section 6.

## 2. Individual Components of an RFID-Based Wireless Sensor System

**Figure 3:**
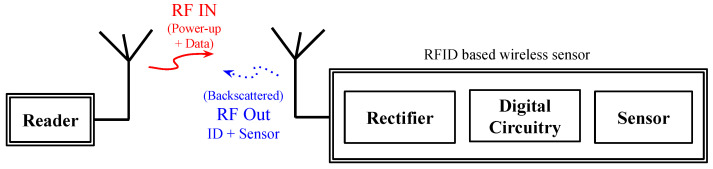
Components of an RFID-based wireless sensor system.

An RFID-based wireless sensor consists of several components. A block diagram of all the key components is shown in Figure 3, and details of each component are discussed below.

### 2.1. Antenna

An antenna is a transducer that converts free space electromagnetic energy to guided electromagnetic energy and vice versa to enable wireless communication in an RFID system. Although any radiating structure can be termed as an antenna, the efficiency with which it can transform the electromagnetic energy plays a major role in determining its amenability for use in sensor communication [29].

To design antennas, certain characteristics are of key importance. These characteristics are the resonant frequency, bandwidth, impedance, gain, radiation pattern, and polarization. Any design is a trade-off of these characteristics and must be optimized based on the application of interest. In an RFID-based sensor, usually small size, planar, and high gain antennas are desired to ensure longer read range and lower fabrication cost [50,51].

To achieve a small size, different miniaturization techniques are used. Amongst them, meander line antennas are of great interest due to their simplistic design [52,53,54,55,56,57,58,59]. In these antennas, the antenna arms are folded to reduce the size and to produce distributed capacitive and inductive reactance that produces a global effect on the antenna impedance. Operating an antenna well below its natural resonance frequency to satisfy space constraints implies that the antenna becomes more difficult to match, and impedance matching directly impacts the RFID read range. Therefore, the reactance must be properly engineered to effectively reduce the size of the antenna [60,61].

Although a significantly smaller size can be achieved with meander line dipole antennas, they are prone to degradation due to nearby objects and cannot be placed directly on metallic surfaces. Therefore, for applications where a wireless sensor is required to be placed on a metallic object, different classes of antennas may be considered. Normally patch antennas have a ground plane attached on one side and can thus be used for this purpose. However, due to their large size at UHF frequencies, modified versions such as fractal and meandered patch antennas are preferred for RFID tags [51,62,63,64,65].

### 2.2. Rectifier

A rectifier in an RFID tag is the main circuit that converts the incident electromagnetic energy received by the antenna into a DC supply voltage. This voltage is required to operate all the internal circuitry of the tag, which includes the analog circuitry, base-band DSP circuitry, and memory of the tag [66,67,68,69,70].

The power efficiency and stability of the rectifier are determining factors for the range of the RFID tag. Generally, Schottky diodes are used in AC/DC rectifiers; however, for RFID tags, they are avoided. This is because they cannot be co-fabricated with CMOS technology, which is required for DSP and memory design. In contrast, separate fabrication of both would result in inconsistencies that degrades the performance of the chip. Therefore, diode-connected MOS FETs are preferred when designing RFID rectifiers as they can be accurately co-fabricated with other components [66,71]. A detailed model incorporating the fabrication process yields rectifiers with optimal performance [72,73,74,75].

If standard threshold voltage CMOS devices are used, the rectifier cannot be turned on when the voltages at its terminals are lower than its turn-on voltage, which affects the read range of the RFID tag. Solutions using near differential-drive rectifier, photovoltaic-assisted rectifier, and zero threshold-based technologies such as Silicon-on-Sapphire and Hetero-junction Tunnel FET provide a significant improvement to the read range [44,76,77,78,79,80,81,82,83,84].

### 2.3. Digital Circuitry

Generally, an RFID tag consists of digital circuitry that is used to transmit the identity data of the chip. This circuitry obtains data from the memory of the tag and modulates it over the backscattered signal. To integrate a sensing element into an RFID tag, an additional digital circuitry that can utilize off-the-shelf sensing elements may be added. This addition allows for recording of sensed data directly inside the tag and for sending it back to the reader using digital modulation techniques along with the identification data. The major benefit of this technique is that the accuracy of the sensor can be very high [23]. However, a clear drawback is that this digital circuity requires additional power. For passive RFID tags, power is limited, and these additional circuits can only operate at the expense of a read-range reduction.

On-chip digitally integrated sensors consist of three major blocks: a digital control circuit, an off-the-shelf sensing element, and an analog-to-digital converter (ADC). The digital control circuitry drives the sensing element by providing the required current or voltages. The element generates an analog voltage based on the physical parameter being measured. This voltage is converted into a digital format using the ADC and transmitted back to the reader using the aforementioned RFID circuitry. Using this technique, different sensors such as temperature, gas, and food-quality sensors can be easily connected [23,34,85,86].

### 2.4. Sensing Element

**Figure 4:**
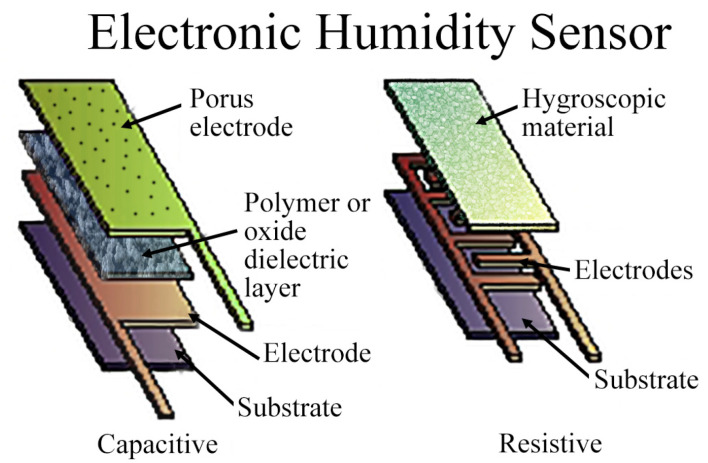
Design comparison of resistive- and capacitive-type humidity sensors.

The sensing element is the heart of the wireless sensor. It is the component that is actually sensitive to the parameter of interest. From this point of view, there are two types of sensing elements: resistive and reactive [87,88,89,90]. Resistive sensing elements are the ones for which the resistance across the terminals changes with variations in the physical parameter being measured. Similarly, reactive sensing elements are usually capacitive or inductive in nature and their reactance varies. Resistive sensing elements are usually lossy as power must be dissipated to read their value. On the other hand, reactive sensing elements, especially the capacitive variety, can be very energy-efficient as very little current is drawn to operate them. A simple structure of resistive and capacitive type humidity sensors is shown in Figure 4 [91].

Based on the layout or packaging design, sensing elements can exhibit certain parasitics. As a result, not all sensing elements can be used at high frequencies, such as those employed in RFID. If an on-tag digital circuitry for the tag exists, generally off-the-shelf sensing elements may be used. However, if the topology being used drives the sensing element using a high-frequency incident signal, then the sensing element must be responsive and sensitive at the frequency being used. In this case, a reader must be able to understand the received information and to separate it from the regular RFID’s identity information. Elements operating at high frequencies are not easily obtainable on the market. As a result, different types of sensing elements for RFID sensors are currently being explored by researchers to increase their frequency of operation while simultaneously reducing their power consumption [34,35,92,93,94,95,96,97,98].

## 3. System Topologies

Different arrangements and utilization of the RFID tag’s components can result in different topologies. There are five principal topologies used, each offering different levels of complexity, cost, read range, and accuracy. Here, the details of each topology are discussed to analyze their pros and cons.

### 3.1. Chip-Less RFID Sensor Topology

**Figure 5:**
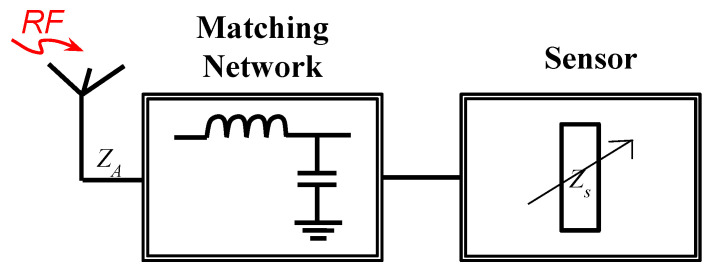
Block diagram of a chip-less RFID-based wireless sensor.

**Figure 6:**
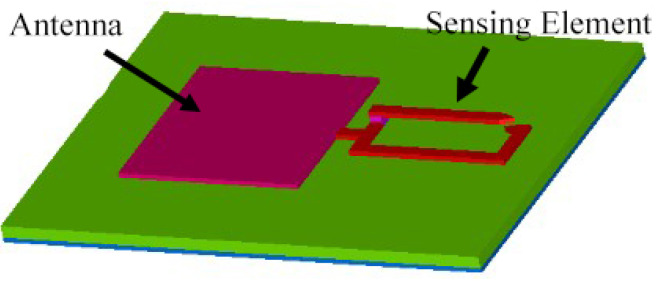
A chip-less RFID-based wireless strain sensor design [30].

The simplest form of RFID sensor requires no integrated circuits (ICs) and communicates sensed data by simply varying the radar cross section (RCS) of the tag at a certain frequency [8,29,30,31,32]. This is achieved by attaching a sensor, having an input impedance of $Z_{S}$, with an antenna, having an input impedance of $Z_{A}$, through a matching network. A block diagram is shown along with an example of a strain sensor in Figure 5 and Figure 6, respectively. RCS is a combination of structural-mode reflection and antenna-mode scattering. An incoming electromagnetic wave is partly absorbed and partly reflected from the surface of the antenna. The signal reflected from the surface is known as a structural-mode reflection, whereas any part of the absorbed signal that is reflected due to impedance mismatches is known as antenna-mode scattering [99,100,101,102]. The sensing element, which could be designed for sensing temperature, humidity, or gas, transforms any change in the physical quantity into a variation in its electrical properties such as resistance or reactance. As a result, the resonance frequency or the quality of the matching network is altered. Hence, this causes a change in the RCS of the antenna at a given frequency.

To read data from a chip-less RFID sensor, a reader transmits a frequency sweep signal of a specific bandwidth and analyzes the backscattered signals that it receives. These backscattered signals are affected by the physical location of the sensor and its RCS. If the physical location and distance between the sensor and the reader are fixed, then the effect of the physical location can be easily factored out to determine the RCS, specifically of the sensor.

When the antenna impedance $Z_{A}$ is matched to the sensing element’s impedance $Z_{S}$, the reflection goes to zero and the RCS drops to a minimum. As the mismatch increases with a change in the impedance of the sensing element, which is affected by the physical condition being sensed, the mismatch between the antenna and the sensing element increases. This eventually increases the RCS of the sensor. As a result, the amplitude of the backscattered signal increases. This increase is directly related to the sensing element and can be easily translated to the change in the physical quantity by using predetermined calibration coefficients.

Another perspective to understand this type of sensor is that, when the sensing element mismatches, the resonance frequency of the circuit shifts and, thus, the sensor is matched to another neighboring frequency. The reader can easily search this resonance by measuring the backscattered signal at different frequencies and by matching it with the predetermined calibration data to determine the sensor value and the corresponding physical quantity [103,104,105,106].

Although the aforementioned is by far the most cost-effective method of RFID sensing, it requires a higher frequency bandwidth and is also prone to multi-path and environmental effects. Moreover, areas having multiple sensors in close proximity are not supported. Therefore, this method is mostly preferred in uncluttered areas such as farms or fields. There are techniques where multiple resonators are used so that data in multiple frequency bands could be analyzed to reduce the effect of multi-path and environmental noise [95]. Moreover, diode frequency doubling is sometimes used in the sensor so that reception at the higher harmonic is free of environmental backscatter, which is high at the actual frequency of transmission [107,108,109]. Another technique is to use a direct conversion six-port network in which information is divided into in-phase and quadrature components. The reference signal for calibration purposes is sent as an in-phase component while the sensing element signal is sent as the quadrature component so that they could be analyzed together to determine the sensed value [110,111]. Nevertheless, these techniques increase the cost and reduce the read range of chip-less wireless sensors. The read range demonstrated by these sensors ranges from 2 m to 30 m [8,29,95,103,104,105,106,110,111]. Moreover, due to multi-path and analog communication, the accuracy of these sensors is low.

### 3.2. Chip-Based RFID Sensor Topology

To address the challenge of multi-path propagation and to support multiple sensors in close proximity, wireless sensors must incorporate digital communication techniques. This is achieved in chip-based RFID tags, where the backscattering is digitally controlled and acts as a digitally modulated signal. This enables assigning a unique identifier to multiple tags in a vicinity, employing anti-collision protocols, and providing error reduction methods. All of these techniques are built into the RFID EPC-Gen2 protocol. Chip-based RFID tags based on this protocol can be modified in several ways to integrate sensors inside them. These modifications generally include an antenna-resonance shifting-based sensor, a multi-port architecture to remove the sensing element from the incoming signal path, a digitally integrated sensor using digital circuitry, and an ambient energy-harvesting block to get additional power from the surroundings.

#### 3.2.1. Chip-Based Antenna Resonance Topology

One of the simplest topologies of chip-based RFID sensors operates on principles that are very similar to chip-less RFID sensors. Generally, the RFID tag is connected to an antenna using some kind of matching network. The matching network has a resonance at a particular frequency. Any change in the reactive or resistive component of the matching network shifts its resonance frequency or alters the loss of the network. As a result, the RCS of the RFID tag varies [33,34,35,36,37,38].

**Figure 7:**
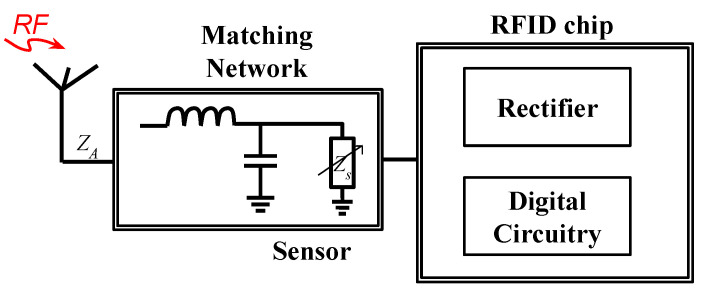
Block diagram of a chip-based antenna resonance wireless sensor.

**Figure 8:**
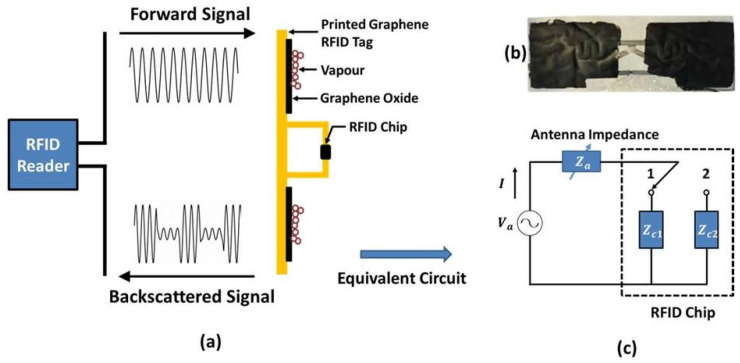
(**a**) Operating principle of the chip-based antenna resonance RFID sensor. (**b**) Printed with a 15 μm thick layer of graphene oxide on top. (**c**) The equivalent circuit of the sensor [112].

To read data from wireless sensors working on the chip-based antenna resonance topology, a reader first sends an interrogation signal. This signal energizes the rectifier circuitry, and when the charging reaches a threshold level, the IC wakes up. The IC backscatters the interrogation signal by shorting and matching its terminal to send binary signals. As a result, the backscattered signal amplitude increases or decreases and the reader determines the high and the low signal. This information is used to demodulate the data being sent by the RFID tag. Since the sensing element influences the matching network of the RFID tag, the backscattered signal is different at different frequencies. The reader can simply sweep the frequency of its interrogation signal to determine the tag’s RCS at different frequencies. By utilizing the digital signal and the amplitude of the backscattered signal at different frequencies, the reader identifies the tag and determines the sensed value, respectively. A block diagram of this topology is shown in Figure 7, and an example of such a sensor is shown in Figure 8.

In practice, the modifications in the matching network due to the changes in the sensing element also affect the quality of the matching network. Therefore, the shifted resonance may not provide maximum power transfer to the RFID tag. As a result, based on the sensor’s dynamic range, the read range, which is directly dependent on the strength of the interrogation signal received by the rectifier circuit, might be lower at some states of the sensor. This will eventually determine the actual read range of the RFID sensor. Moreover, since the sensor information is delivered through analog communication, only moderate accuracy is obtained. Furthermore, the shift in resonance requires a higher bandwidth for the sensor to operate. In contrast, the design is low cost as the sensor is passively integrated into the matching network. Chip-based RFID antenna resonance wireless sensors have been proposed operating within the read range of 3 to 5 m [33,34,35,36,37].

#### 3.2.2. Chip-Based Multi-Port Topology

Keeping the read range of an RFID tag intact and unaffected by the addition of a sensing element is one of the main goals of research on RFID-based wireless sensors. Usually, the power-up signal reaching the RFID chip is the limiting factor in the read range of an RFID-based wireless sensor. As the reader is connected to a power source, it can provide a good amount of power. However, the effective isotropic radiated power (EIRP) is limited to 36 dBm by the standards of RFID EPC-GEN 2 and cannot be increased above this level. On the other hand, the tag must also receive the minimum amount of power required to generate enough voltage to operate its circuitry. Therefore, any losses in the sensing element affect the read range of the RFID tag.

**Figure 9:**
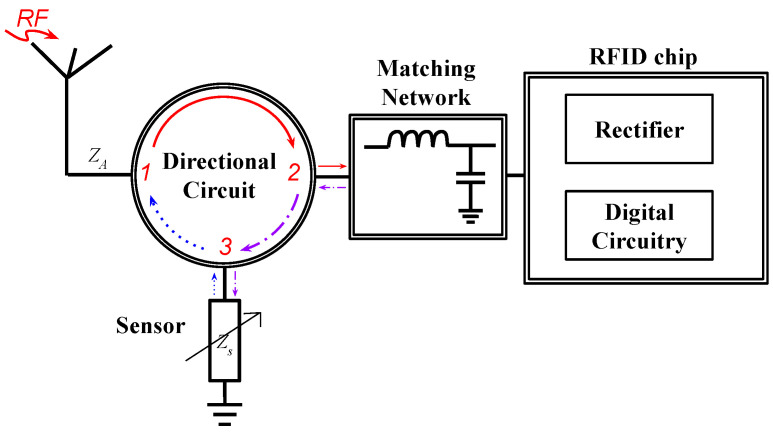
Block diagram of a chip-based multi-port wireless sensor.

**Figure 10:**
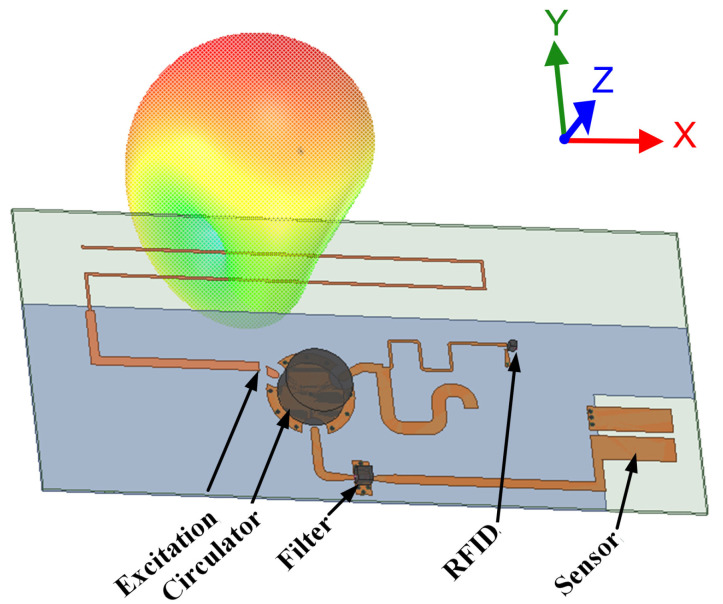
Three-dimensional model of a chip-based multi-port wireless sensor [113].

To ensure that the power-up signal reaches the RFID tag’s rectifier without incurring any loss, there should be no lossy component added in its path. In the previous topology, we observed that the signal passing through the matching network undergoes some loss as the sensed value changes. To address this issue, another topology was recently proposed in which the sensing element was removed from the path of the signal and attached to the path of the backscattered signal. This was achieved by using a multi-port device that separates the incoming signal from the backscattered signal. A block diagram of this topology and a 3D plot of a sample design are shown in Figure 9 and Figure 10, respectively.

This topology ensures that the power-up signal reaches the RFID tag without any significant loss. The backscattered signal is sent to the sensing element where an additional phase delay is introduced. The sensor information in the phase can be easily extracted by the reader. Although having sensor circuit introduces extra loss in the backscattered signal, the reader is connected to a power source and can interpret and demodulate a fairly low-power signal. Therefore, this topology provides an improvement in terms of read range compared to the previous one in Section 3.2.1 simply by eliminating the sensing element from the power-up signal path. Since the sensor information is a hybrid of digital and analog communication, the required bandwidth is low. However, having a multi-port device requires components that may increase the cost by a few dollars. Sensors with medium accuracy operating at a range of 7 m have been reported in [39,40,41].

#### 3.2.3. Chip-Based Digitally Integrated Topology

Another chip-based RFID topology is one in which the sensing element is attached to digital circuitry, which reads its value and sends the data digitally back to the reader [114]. To accomplish this, the incoming interrogation signal first energizes the rectifier circuit. After the required threshold level is achieved, the added digital circuitry in the IC uses a portion of this power to determine the sensed value.

**Figure 11:**
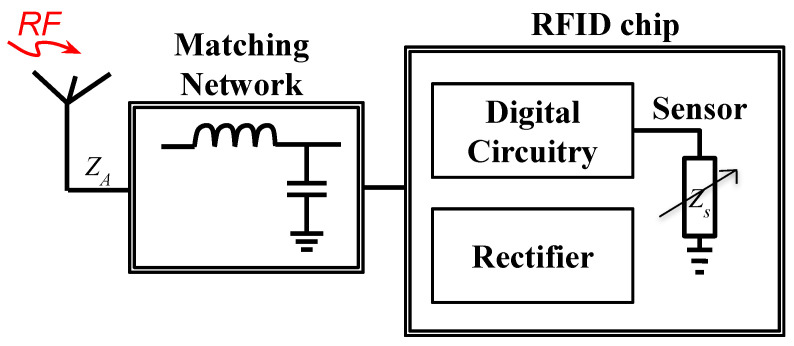
A simple block diagram of a chip-based digitally interfaced sensor.

**Figure 12:**
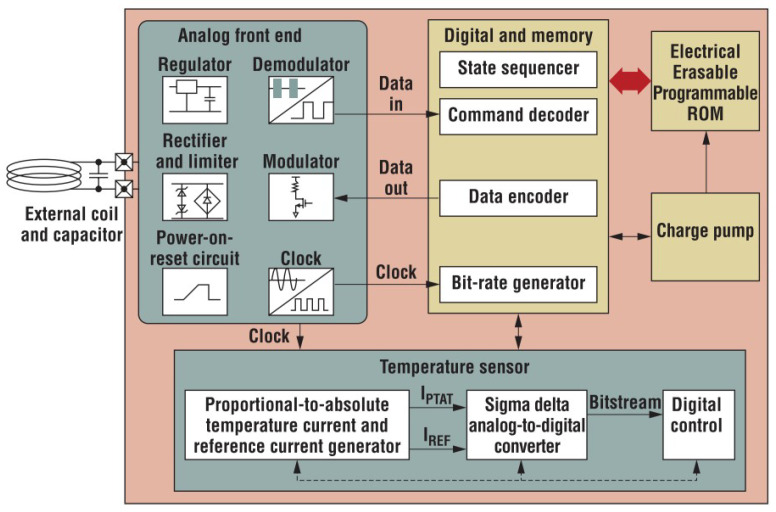
A chip-level block diagram of a chip-based digitally interfaced RFID sensor [23].

One of the methods to design the digital circuitry is to have a phase-locked loop (PLL)-based sensor interface that compares the phase of two oscillators, where one of the oscillators is connected to a reference capacitor and the other is attached to the capacitive sensing element. Any change in the element introduces a phase difference between the two oscillators. This change is determined through the error port of the PLL. The amplitude of the error signal is directly related to the difference between the capacitance value of the reference capacitor and the element. The error signal is read by an on-chip ADC, which is then digitally concatenated to the tag’s identity data and sent back to the reader through the backscattered signal [43]. A simple block diagram and a chip-level block diagram of the digitally integrated sensor are shown in Figure 11 and Figure 12, respectively. The chip level block diagram includes details of a digitally integrated temperature sensor, which shows what components are required to design the sensor.

The main challenge in a chip-based digitally integrated sensor is having digital circuitry that operates at very low voltage and uses a minimum amount of power to read the sensed value with a suitable accuracy. This added circuitry can significantly reduce the read range of an RFID chip and can slightly increase its cost. Currently, sensors utilizing this topology have been demonstrated with read ranges of around 0.7–2.2 m [42,43]. It should be noted that, since the sensor information is communicated digitally, the accuracy is high and the bandwidth is the same as a regular RFID chip.

#### 3.2.4. Chip-Based Ambient Energy-Harvesting Topology

**Figure 13:**
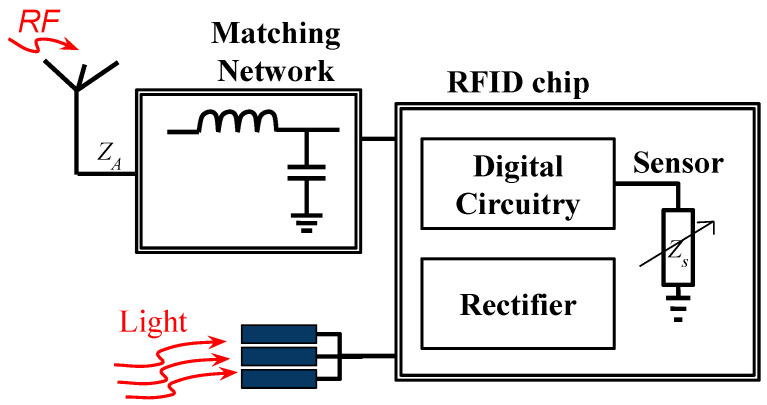
Block diagram of a chip-based ambient (light) energy-harvesting wireless sensor.

**Figure 14:**
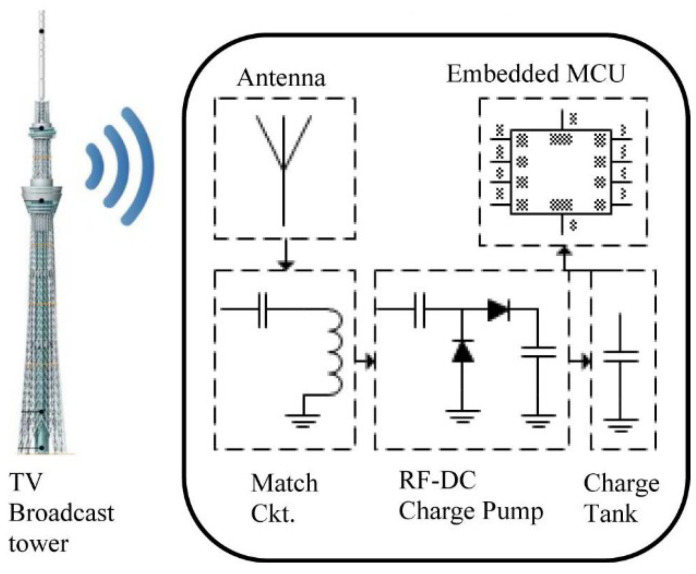
Prototype of a chip-based ambient (RF) energy-harvesting wireless sensor [45].

To improve the read range of chip-based RFID sensors without adding a battery, harvesting the energy from an ambient source can be of help. There are multiple power sources that are ambient—solar power and indoor lights or RF signals from radio and TV stations, WiFi networks, and cellphone towers [44,45,46,47]. By attaching a small solar cell and/or by using a wide-band receiver that is capable of receiving RF signals from an ambient source, the overall energy received by an RFID-based wireless sensor can be tremendously increased. Therefore, without adding any battery, the read range of the sensor can be improved. A block diagram and a prototype of such a sensor tag are shown in Figure 13 and Figure 14, respectively.

If the RFID chip operates on the principles of backscattering the transmitted signal, the limiting factor, in this case, is the reader’s sensitivity, as follows. In RFID communication, a power-up signal faces two-way path loss and any losses inside the RFID tag, which can be antenna mismatch, sensing element loss, and RFID backscattering switch loss. In an ideal case where there is no power lost inside the tag and considering the maximum allowed EIRP of 36 dBm, with a receiver sensitivity of −90 dBm and a tag antenna gain of 3 dB, the two-way path loss at 915 MHz allows for 66 dB path loss in one direction. This corresponds to around 52 m of distance between the reader and the tag. Here, we considered that the RFID tag is obtains enough energy from an ambient source to fully power-up its internal circuitry and the digital circuitry of the sensor. Sensors utilizing this topology, using only solar cells, have shown a read range of around 15 m [20]. Since the sensor information is sent digitally, the accuracy is high. Furthermore, the bandwidth is the same as a regular RFID chip, whereas the cost increases due to the added solar panels.

### 3.3. Topology Summary

A comparison of all five aforementioned RFID-based sensor topologies is presented in Table 1. The sensors are compared against their cost, complexity, bandwidth, anti-collision support, dense deployment support, and range. Each type comes with strengths and weaknesses. If a low-cost solution in an uncluttered environment is required, chip-less-based RFID wireless sensors are an excellent solution. However, if multiple parameter sensing measurements in a highly dense area are required, chip-based solutions may be preferred. Topology in the chip-based solution may be selected based on cost and read-range requirements.

## 4. Implementation and Testing

The theory of five RFID-based wireless sensor topologies has been discussed in detail in the previous section. There are different methods to implement these topologies. Here, we discuss a practical implementation of each. Each example is considered based on its complexity, cost, read range, and accuracy.

### 4.1. Chip-Less RFID Sensor Implementation

#### Example of a Chip-Less RFID Humidity Sensor

Humidity is one of the key elements that is required in controlled amounts in different environments. In homes, extreme dryness can cause deterioration to human skin, whereas for plants and animals, it determines whether they can thrive in the environment. Moreover, it is an important factor that determines if specific electronics and electrical equipment are safe to be used in a given environment, as very high humidity levels may cause a short circuit. Therefore, humidity is an important parameter that needs to be gauged.

Humidity is often measured as Relative Humidity (RH), which is a ratio of the partial pressure of water vapors available in the air to the saturation vapor pressure of the air. To determine this ratio, polymers that absorb water molecules, such as Polyvinyl Alcohol (PVA), are used as a sensing element on a capacitor. As the vapor pressure increases, the polymer absorbs more and more water. As a result, the average permittivity of the capacitor increases, which eventually raises its capacitance.

**Figure 15:**
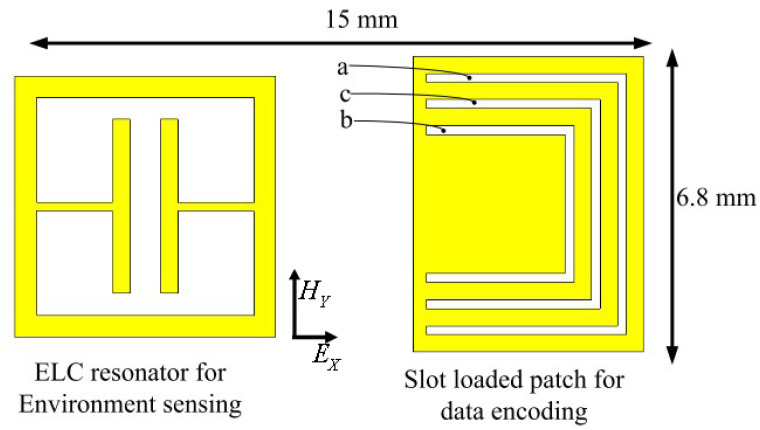
The layout of the chip-less RFID tag humidity sensor [95].

**Figure 16:**
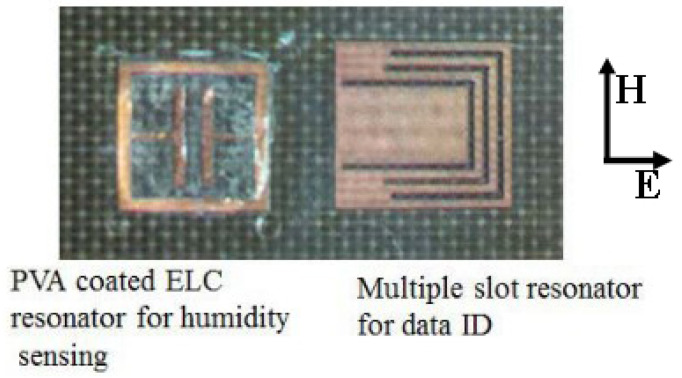
Fabricated circuit of the chip-less RFID tag humidity sensor [95].

To form a chip-less RFID-based wireless humidity sensor, Amin et al. in [95] combined a simple multiple slot resonator with a PVA coated ELC resonator. The multiple slots in the design are used to obtain resonance at different frequencies so that the sensor node could be identified. This was achieved by designing slots of different lengths on a patch without a ground plane. The ELC resonator was separately coated with PVA so that only its resonance changes with the change in the humidity. The layout and fabricated circuit are shown in Figure 15 and Figure 16, respectively.

**Figure 17:**
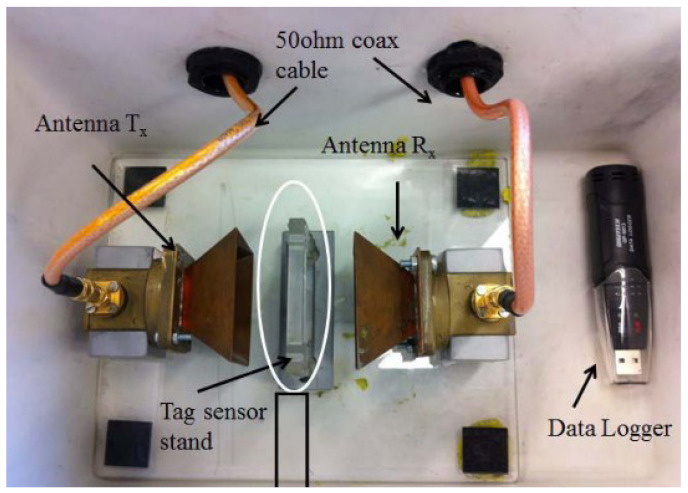
The test setup of the chip-less RFID tag humidity sensor [95].

To evaluate the design, tests were performed in a humidity-controlled chamber. Two horn antennas connected to a Vector Network Analyzer (VNA) were used to study the resonance at varying humidity levels. The wireless sensor was placed between the two antennas, and a frequency sweep was performed using the VNA. The setup is shown in Figure 17.

**Figure 18:**
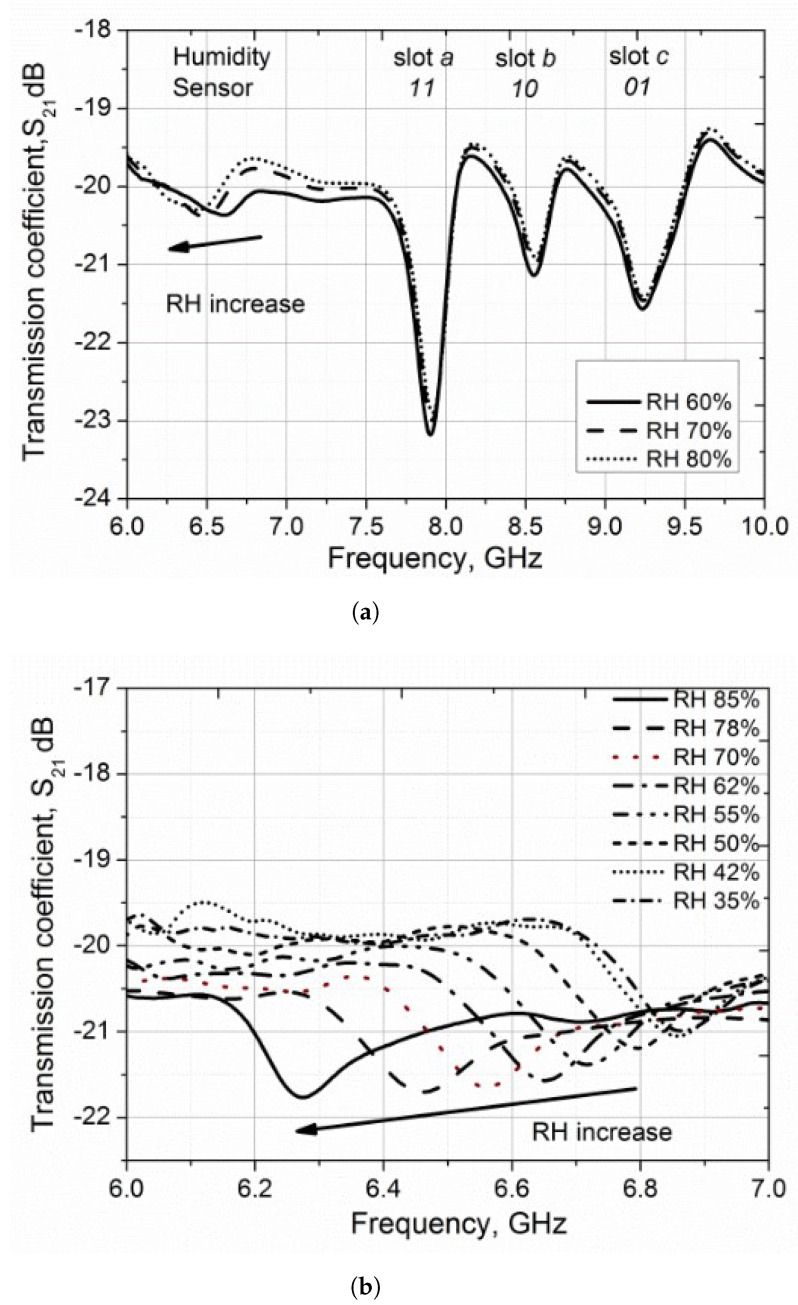
(**a**) Measured transmission coefficient (calibrated) (S21) versus frequency for the chip–less RFID humidity sensor with PVA coating. (**b**) Detailed experimental results with ELC resonator as a humidity sensor [95].

The complex transmission coefficient (S21) at different humidity levels was recorded, and the data are shown in Figure 18a. Three resonance points occurring due to the slots a, b, and c are labeled with their corresponding resonances that help in identifying the tag. It can be seen that these resonances are little affected by the change in humidity. However, the resonance of the sensor, which is around 6.5 GHz, varies with the change in humidity levels. A detailed plot of the sensor response is shown in Figure 18b.

The example combines chipless identification and sensing together. Due to its passive nature, the cost is very low. Moreover, since there is no chip required in the sensor, the read range depends on the reader and clutter around the sensor. Hence it can easily go up to a few meters. However, the accuracy deteriorates as the signal level reaches close to the noise level of the reading device.

### 4.2. Example of a Chip-Based Antenna Resonance

Body movement monitoring is an important observation in medical treatments, athletics, or the army. In medical treatment, monitoring may be required where a patient has undergone surgery and must stretch their body parts to enhance muscle formation. Doctors need to determine the extent of the body movement of their patients. This is achieved by attaching sensors to the patients’ bodies that help to monitor their movements.

In [115], Mehmood et al. proposed an RFID-based passive strain sensor design that is easily integrated into clothing. This integration allows for monitoring strain on the sensor wirelessly. As a result, movements of different parts of the body are easily determined without attaching any wires, which may otherwise introduce hindrance to the mobility of the patient.

**Figure 19:**
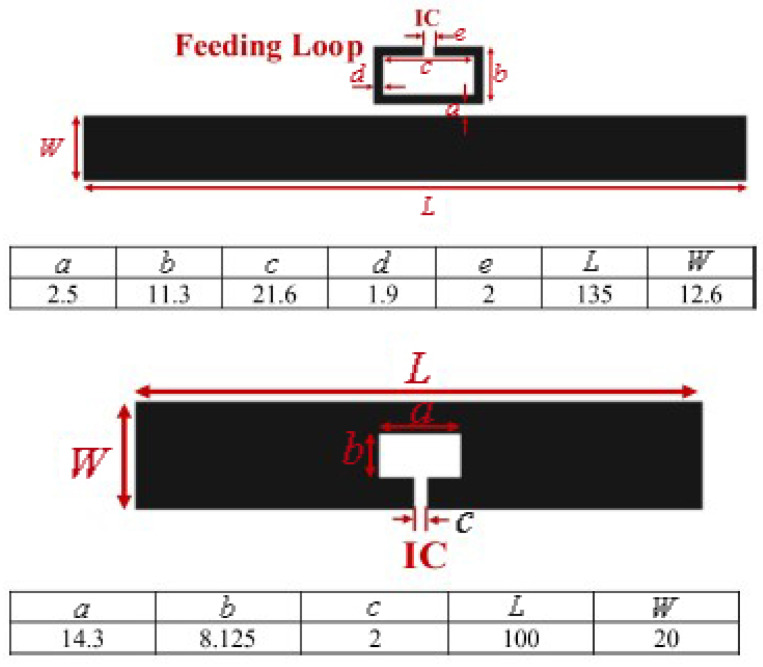
The layout of a chip-based antenna resonance controlled strain sensor [115].

**Figure 20:**
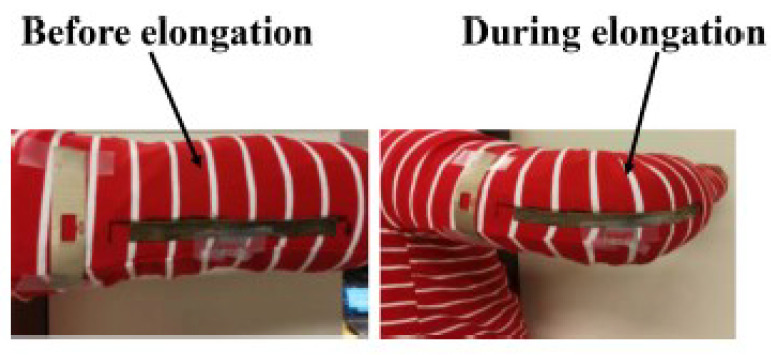
Implemented circuit of the strain sensor on a T-shirt [115].

To design a passive strain sensor, a stretchable antenna was designed so that its resonance changes as it is stretched along its length. The antenna was designed using a conductive textile material, which was easily integrated into a shirt. Moreover, a non-stretchable antenna was also designed and attached to the shirt to act as a reference. The model and fabricated circuits are shown in Figure 19 and Figure 20, respectively.

**Figure 21:**
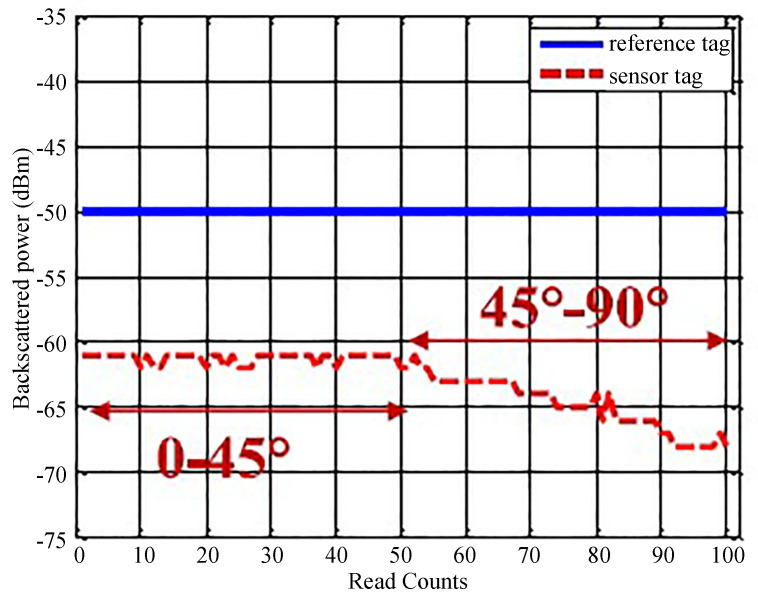
The results of a strain sensor showing varying RCS at different strain levels [115].

To measure the sensed value, a general RFID reader was used that reads the ID of the tag and the amplitude of the backscattered signal. The simultaneous reading of backscattered signals of the reference tag and the sensitive tag allows for determining the sensed value by calculating the difference in their RCS. The results in Figure 21 show the amplitude of the backscattered signal for a straight and a bent arm.

The results show how a simple chip-based antenna resonance varying sensor can be used to determine the stress on a wearable item that could eventually help determine the body movement of a patient. The cost is low since it only requires a single RFID chip, which costs a few cents. The reported range of this wireless sensor in a working environment is around 1 m. The deterioration is mainly because of the losses in the sensor, which directly affect its read range. The accuracy is low because the magnitude of the backscattered signal can be easily affected by ambient noise.

### 4.3. Example of a Multi-Port Chip-Based Battery-Less

Liquid level detection is extremely useful in a wide variety of applications. For example, in houses, a timely warning of flooding can help avoid huge losses. Additionally, in medical treatments, patients are given medications for which determining the rate or amount of the dose that is being delivered to the patient is an important parameter. Using a battery-less and wireless liquid level detector can be very helpful in both applications.

Different fluids have a different amount of conductivity. Using this nature of fluids, liquid levels can easily be determined by making simple probes that are either conductive or capacitive. As the water level rises, the conduction or capacitance of the sensing element varies. In [39,41], Khalid et al. used similar electrodes for a sensing element in which the conduction and capacitance varied with the change in the liquid levels.

**Figure 22:**
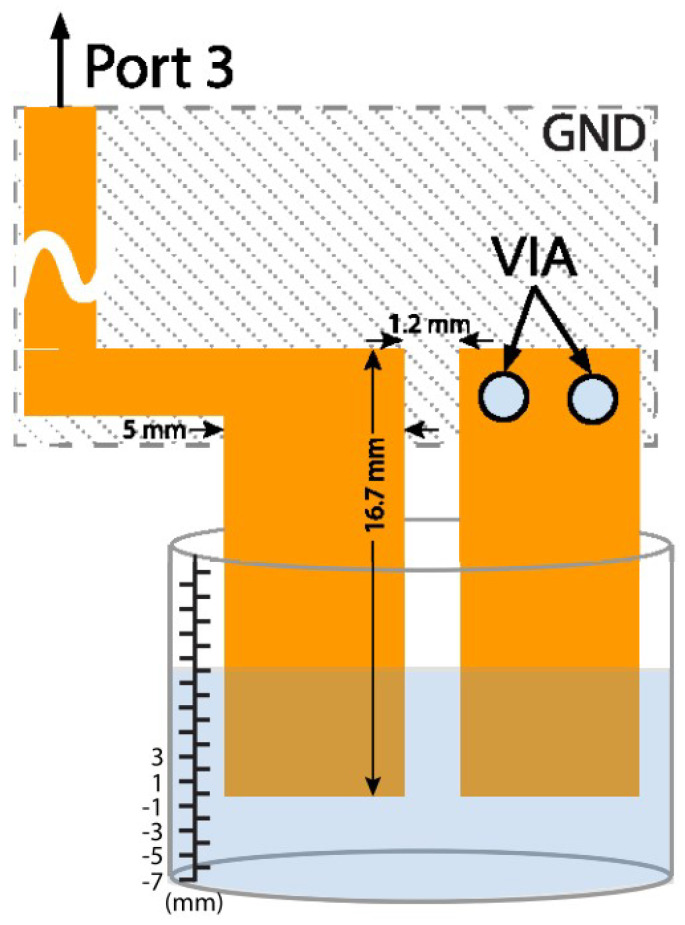
The layout of chip–based multi–port flood sensor [39].

**Figure 23:**
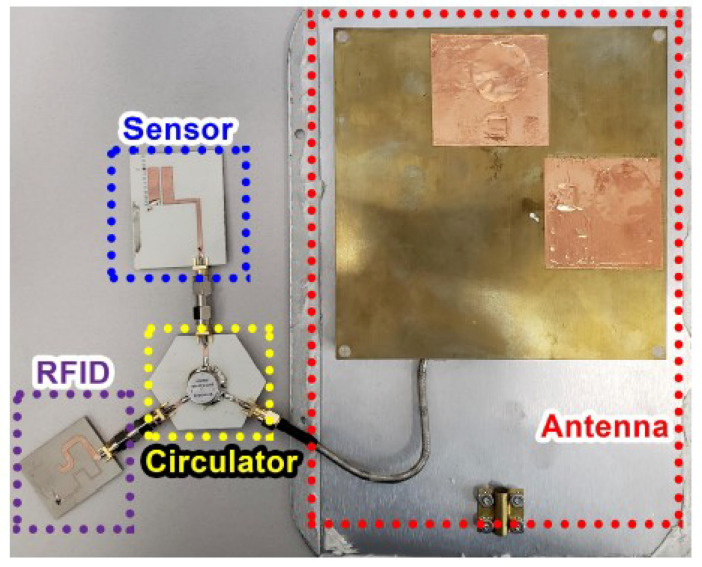
Implemented circuit of the flood sensor [39].

**Figure 24:**
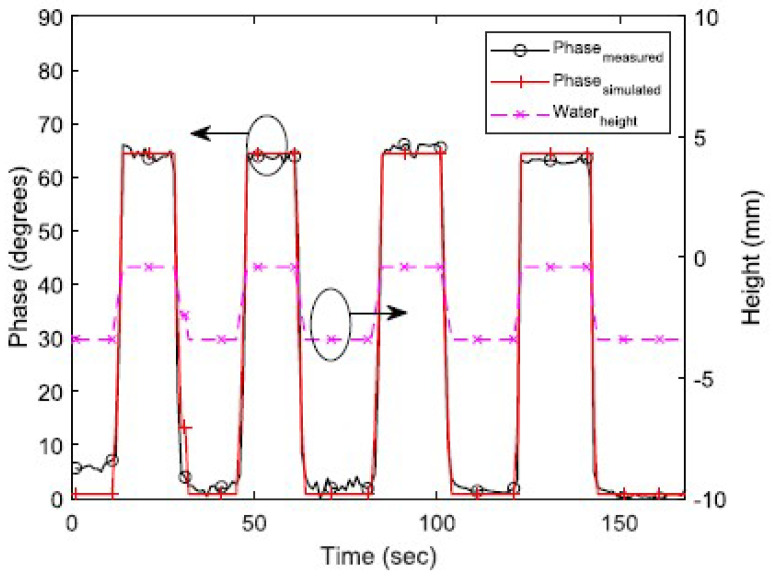
Results of the flood sensor showing varying phases of the backscattered signal at different water levels [39].

Using a multi-port chip-based passive wireless sensor, the incoming signal from the RFID reader is directly sent to the RFID tag. When the tag responds by backscattering the incoming signal, the backscattered signal is routed to the flood sensing element using a simple circulator. The model and circuit are shown in Figure 22 and Figure 23, respectively. The attached element adds a phase delay to the backscattered signal based on its capacitance, which depends on the liquid level inside the container. This delay is easily determined by using a reader IQ-based demodulator. The results in Figure 24 show how the phase of the backscattered signal changes as the level of fluid increases or decreases with time.

Since a circulator and an RFID chip are required, the cost of this design is moderate. The incoming signal reaches the tag without any loss from the sensing element; therefore, the RFID tag’s read range remains unaffected by the addition of the element. The demonstrated range of this sensor was 7 m. The sensor information is sent through phase modulation, and the accuracy is in the medium range.

### 4.4. Example of a Chip-Based Digitally Integrated

In all of the aforementioned topologies, the sensing element integrated into an RFID tag must be responsive at the frequency of operation. Usually, sensors available in the market operate up to a few kHz. The ability to use off-the-shelf sensors can be very helpful where wireless range is not a concern. Moreover, there is a wide range of sensors, such as temperature sensors and force sensors, that operate at low frequencies and are easily available in the market. In [42], Fernandez et al. demonstrated the use of RFIDs that digitally integrate sensors by combining a force sensor and by using an in-built temperature sensor.

**Figure 25:**
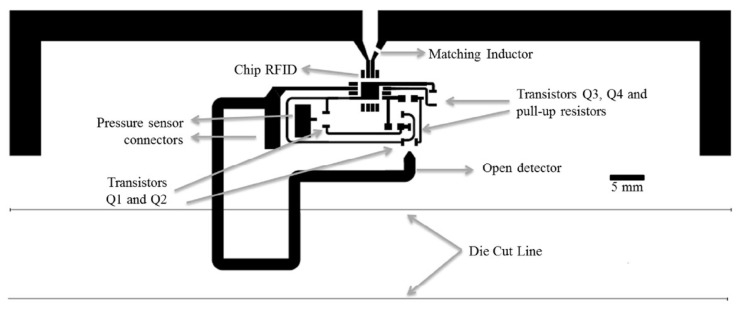
Layout of a chip–based digitally integrated force sensor [42].

**Figure 26:**
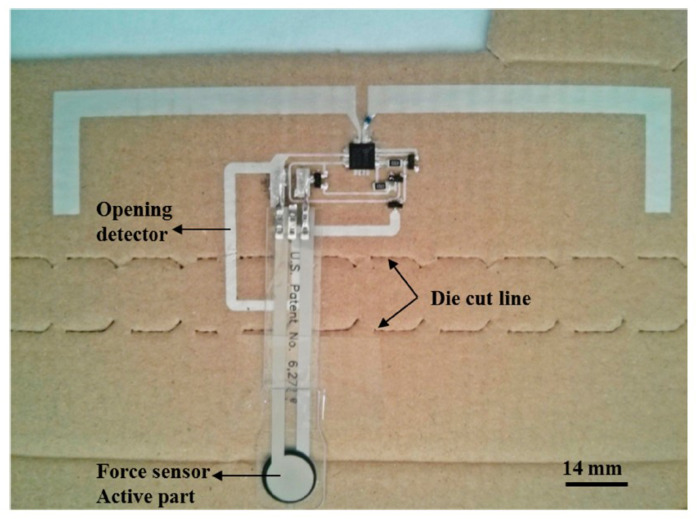
Implemented circuit of the force sensor [42].

The RFID tag used for the demonstration was an SL900A RFID chip (AMS AG, Austria), which is compatible with EPC-Gen 2 RFID standard [116]. This RFID tag incorporates an on-chip A/D converter and a voltage supply. These components can be used to determine the attributes of an external sensor to record its value. This value is digitally sent back to the reader. The designed circuit layout and the fabricated circuit are shown in Figure 25 and Figure 26, respectively.

**Figure 27:**
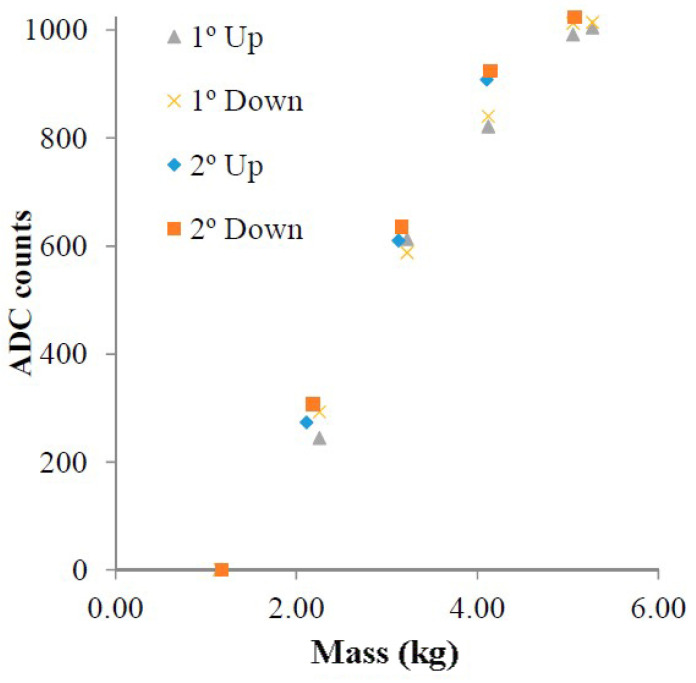
The results of a force sensor showing varying ADC count transmitted by the sensor [42]. X° up/down shows X kg change in weight to observe the sensor’s hysteresis.

The built-in temperature sensor provides a resolution of around 0.23 °C and can be used from −89.3 °C to 147.9 °C. To analyze the performance of the external sensor ports, a force sensor was attached to a container. The force sensor presents a resistance in the range of MΩ when there is no mass attached to it, whereas its resistance decreases as the mass increases. The sensor was tested with weights spanning the range from 1 kg to 4 kg. Multiple values were recorded with single and dual setup increments and decrements to observe the hysteresis of the sensor. A 3% error was found, which was within the error of the force sensor. The results are shown in Figure 27.

Although the design can be simple and cost effective, the use of an additional ADC to record the sensor value results in a huge amount of power being sacrificed in this case. Therefore, when working as a battery-less wireless sensor, the maximum range reported for this RFID-based wireless sensor is only 1.1 m. However, the accuracy is high because the data is sent to the reader through digital modulation techniques.

### 4.5. Example of Chip-Based Ambient Energy Harvesting

All of the previously discussed wireless sensors harvested energy using power that was delivered from the interrogator. Therefore, their range is limited, and they perform well only in short- to mid-range applications. To improve the range, energy harvesting from an ambient source can also be introduced. There are photovoltaic (PV) and non-PV energy sources that are ambient and that can be used for energy harvesting.

With the widespread availability of PV energy through indoor and outdoor lights, PV-based energy harvesting is a very promising technique. In [20], a PV-based RFID sensor that can detect temperature and transmit the information wirelessly has been demonstrated. In this design, an EM 4325 IC from EM Microelectronics was used to incorporate a built-in temperature sensor connected to the internal sensor controller that converts the sensed value from analog to digital. This digital value is communicated back to the reader through backscattering.

**Figure 28:**
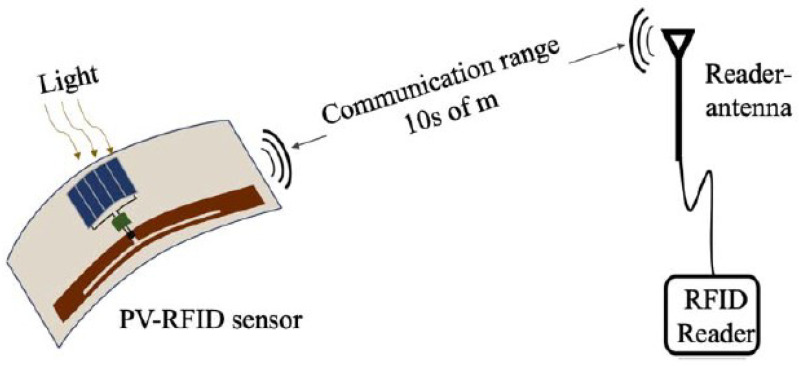
Model of the chip-based ambient energy-harvesting temperature sensor [20].

**Figure 29:**
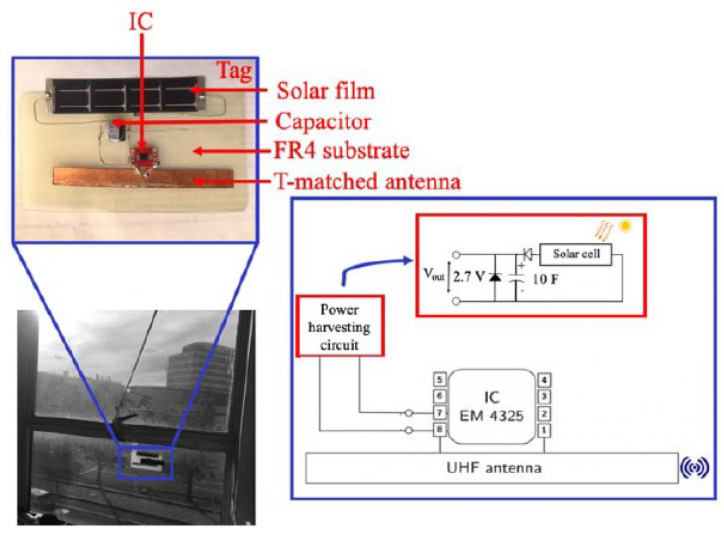
Implemented circuit of the temperature sensor [20].

**Figure 30:**
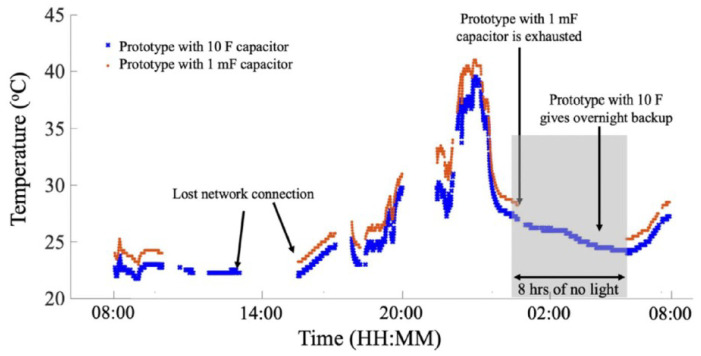
Results of the temperature sensor showing varying temperature and performance recorded for one full day [20].

The RFID tag used in this design allows for connecting an external battery to operate it as an active sensor. However, since the goal is to have a battery-less wireless sensor, PV cells were attached to these ports. A supercapacitor of 10 F was also attached in parallel to the PV cells to harvest the received energy. This increased the cost of the sensor. The model and the fabricated circuit are shown in Figure 28 and Figure 29, respectively. Although the communication/tracking range of up to 18 m was shown, the maximum recorded range, after enabling the integrated sensor, was around 6 m. The results are shown in Figure 30. High accuracy was achieved as the sensor data is digitally communicated back to the reader.

## 5. Future Directions

Although a vast amount of research has already been carried out on battery-less RFID-based wireless sensors, it is clear that a great deal of potential remains for future discoveries. Among the several sensor parameters discussed in this review (e.g., read range, accuracy, cost, and size), it is evident that an improvement in sensor read ranges is still of prime interest to the community. By looking at the aforementioned topologies, we can deduce that a combination of chip-based multi-port and ambient energy harvesting can yield a much higher range—theoretically, up to 50 m.

We also saw that the size and cost of the multi-port topology are not optimal but may be significantly improved through the use of highly miniaturized antennas employing novel matching techniques to enable compact, long-range RFID-based battery-less wireless sensors [60,61].

If accuracy is a concern, digitally integrated sensor topologies with ambient energy harvesting show a great deal of promise. To increase the read range, ambient PV and RF energy may be combined. Moreover, the fabrication of the rectifier circuitry must be engineered to achieve better results. This involves using detailed models of the fabrication process that produce more accurate results and higher consistencies between different batches.

Lastly, we observed that there is a scarcity of sensor components operating in the low GHz range. Research that seeks high-frequency sensing component designs is also needed. This will allow RFID-based battery-less sensors to be used in many new applications, readying them for deployment in the future Internet-of-Things.

## 6. Conclusions

An in-depth review of battery-less RFID-based wireless sensors was conducted. All of the different components of an RFID sensor, including the antenna, rectifier, digital circuit, and sensing element, were discussed in detail. Various topologies that use the components in different ways were investigated. It was observed how the utilization of the limited power and arrangements of the components can affect the read range of a battery-less RFID-based wireless sensor. Real-world implementations of humidity, flood, force, and temperature sensors were discussed. It was noted that chip-based topologies provide the maximum read range with dense deployment capabilities. Finally, some potential future directions for battery-less RFID-based wireless sensors were presented.

## Figures and Tables

**Table 1:** Comparison of the five RFID-based sensor topologies discussed.

Parameter	Chip-Less [8,29,95,103,104,105,106]	Antenna Resonance [33,34,35,36,37]	Multi-Port Architecture [39,40,41]	Digitally Integrated [42,43]	Ambient Energy Harvesting [20]
Cost	Very Low	Low	Moderate	Moderate	High
Complexity	Very low	Moderate	Low	Low	Moderate
Bandwidth required	∼MHz	∼MHz	∼kHz	∼kHz	∼kHz
Anti-collision	No	Yes	Yes	Yes	Yes
Dense deployment	No	Yes	Yes	Yes	Yes
Range (m)	2–30	3–5	7–10	1–2	5–10

## Data Availability

Not applicable.
